# Emerging of a SARS-CoV-2 viral strain with a deletion in nsp1

**DOI:** 10.1186/s12967-020-02507-5

**Published:** 2020-08-31

**Authors:** Francesca Benedetti, Greg A. Snyder, Marta Giovanetti, Silvia Angeletti, Robert C. Gallo, Massimo Ciccozzi, Davide Zella

**Affiliations:** 1 Institute of Human Virology, School of Medicine, University of Maryland, Baltimore, USA; 2 Department of Biochemistry and Molecular Biology, University of Maryland, Baltimore, USA; 3 Department of Microbiology and Immunology, University of Maryland, Baltimore, USA; 4 Flavivirus Laboratory, Oswaldo Cruz Institute, Oswaldo Cruz Foundation, Rio de Janeiro, Brazil; 5 Medical Statistic and Molecular Epidemiology Unit, University of Biomedical Campus, Rome, Italy; 6 Department of Medicine, University of Biomedical Campus, Rome, Italy; 7 Global Virus Network, Baltimore, USA

**Keywords:** SARS-CoV-2, COVID-19, nsp1, Deletion, Pathogenic, Viral adaptation

## Abstract

**Background:**

The new Severe Acute Respiratory Syndrome Coronavirus-2 (SARS-CoV-2), which was first detected in Wuhan (China) in December of 2019 is responsible for the current global pandemic. Phylogenetic analysis revealed that it is similar to other betacoronaviruses, such as SARS-CoV and Middle-Eastern Respiratory Syndrome, MERS-CoV. Its genome is ∼ 30 kb in length and contains two large overlapping polyproteins, ORF1a and ORF1ab that encode for several structural and non-structural proteins. The non-structural protein 1 (nsp1) is arguably the most important pathogenic determinant, and previous studies on SARS-CoV indicate that it is both involved in viral replication and hampering the innate immune system response. Detailed experiments of site-specific mutagenesis and in vitro reconstitution studies determined that the mechanisms of action are mediated by (a) the presence of specific amino acid residues of nsp1 and (b) the interaction between the protein and the host’s small ribosomal unit. In fact, substitution of certain amino acids resulted in reduction of its negative effects.

**Methods:**

A total of 17,928 genome sequences were obtained from the GISAID database (December 2019 to July 2020) from patients infected by SARS-CoV-2 from different areas around the world. Genomes alignment was performed using MAFFT (REFF) and the nsp1 genomic regions were identified using BioEdit and verified using BLAST. Nsp1 protein of SARS-CoV-2 with and without deletion have been subsequently modelled using I-TASSER.

**Results:**

We identified SARS-CoV-2 genome sequences, from several Countries, carrying a previously unknown deletion of 9 nucleotides in position 686-694, corresponding to the AA position 241-243 (KSF). This deletion was found in different geographical areas. Structural prediction modelling suggests an effect on the C-terminal tail structure.

**Conclusions:**

Modelling analysis of a newly identified deletion of 3 amino acids (KSF) of SARS-CoV-2 nsp1 suggests that this deletion could affect the structure of the C-terminal region of the protein, important for regulation of viral replication and negative effect on host’s gene expression. In addition, substitution of the two amino acids (KS) from nsp1 of SARS-CoV was previously reported to revert loss of interferon-alpha expression. The deletion that we describe indicates that SARS-CoV-2 is undergoing profound genomic changes. It is important to: (i) confirm the spreading of this particular viral strain, and potentially of strains with other deletions in the nsp1 protein, both in the population of asymptomatic and pauci-symptomatic subjects, and (ii) correlate these changes in nsp1 with potential decreased viral pathogenicity.

## Background

Severe Acute Respiratory Syndrome Coronavirus-2 (SARS CoV-2) belongs to the realm *Riboviria*, order *Nidovirales*, suborder *Cornidovirineae*, family *Coronaviridae*, subfamily *Orthocoronavirinae*, genus *Betacoronavirus* (lineage B), subgenus *Sarbecovirus*, and the species *Severe acute respiratory syndrome*-*related coronavirus*, and is the virus responsible for the current global pandemic [1–3]. The genome of SARS-CoV-2 [4] is highly homologous to the coronavirus that caused the SARS epidemic in 2003, SARS-CoV [5, 6] and to the coronavirus responsible for the Middle-Eastern Respiratory Syndrome, MERS-CoV [7].

Coronavirus Diseases (COVID-19) comprises symptoms reported by patients infected by SARS-CoV-2, ranging from mild to severe, and some cases result in death. Severe acute respiratory illness with fever and respiratory symptoms, such as cough and shortness of breath, are the primary case definition, but recently patients without respiratory symptoms are becoming more recognized, with manifestations such as gastrointestinal, olfactory, cardiovascular, and neurological. Cases resulting in death are primarily middle-aged and elderly patients with obesity and/or pre-existing diseases (tumor surgery, cirrhosis, hypertension, coronary heart disease, diabetes, and Parkinson’s disease) [8–11].

Given the similarity among the viruses, the data about biological functions, characteristics and effects on the host of the proteins expressed by SARS-CoV-2 are mostly inferred by the previous studies on SARS-CoV and other related human (e.g. MERS-CoV) [12–14] and animal coronaviruses (e.g. mouse hepatitis virus) [15]. In SARS-CoV two large polyproteins, ORF1a and ORF1ab, are cleaved by a specific protease to form 16 nonstructural proteins (nsp), four structural proteins, namely spike (S), envelope (E), membrane (M), and nucleocapsid (N), and eight accessory proteins: ORF3a, ORF3b (absent in SARS CoV-2), ORF6, ORF7a, ORF7b, ORF8a, ORF8b, and ORF9b (absent in SARS-CoV-2). Experimental data indicate that some accessory proteins are considered not essential for viral replication, while others have been demonstrated to be important for virus-host interactions both in vitro and in vivo [16, 17].

Among these proteins, SARS-CoV, nonstructural protein 1, nsp1 also known as the leader protein, plays a central role in hampering the anti-viral innate immune response, in particular Interferon-alpha expression [18], and it has been considered as a possible target for therapeutic interventions aimed at reducing viral pathogenicity [19]. Further indicative of its preserved biological function, nsp1 from alpha- and beta-CoVs have different size, but show comparable biological activities in their ability to reduce host gene expression, even though the mechanism seems different [15, 20–22].

SARS-CoV nsp1 almost completely blocks host protein translation by binding the 40S ribosome of the host cell, which stops canonical mRNA translation at different steps during the initiation process [23–25]. This in turn results in template-dependent endonucleolytic cleavage, followed by degradation of mRNAs of infected cells, while viral mRNA shutdown is avoided through a still not clear mechanism involving interaction between nsp1 with a conserved 5′ untranslated region of the SARS-CoV mRNA [26]. By blocking expression of several components of the innate immune system, including the interferon response, SARS-CoV is thus able to maintain viral expression and escape immune system detection [21].

Critical for this mechanism are certain amino acid residues of nsp1. For example, in the case of SARS-CoV several residues have been identified that differentially inhibit host gene expression, like interferon alpha, responsible for antiviral activity [18]. More recently, a region in the C-terminal domain of nsp1 of SARS-CoV-2 has been demonstrated to interfere with host expression factors [25].

Here we describe a deletion identified in the C-terminal region of nsp1 observed in certain genomes from SARS-CoV-2 patients, from different areas of the word. The deletion did result in removal of three amino acid residues (KSF). Two of them (KS) have been shown to be responsible for nsp1 of SARS-CoV partial attenuation of both inhibition of signal transduction and inhibition of gene expression, including Interferon-alpha [18]. Our data indicate that a small percentage of SARS-CoV-2 viruses is actually harboring a deletion in an important protein responsible for pathogenesis, possibly adapting toward a decrease pathogenicity.

## Methods

We analyzed 17,928 genomic sequences obtained from the GISAID database (updated on 07/24/2020) derived from patients infected by SARS-CoV-2 from different areas around the world. The genomes were collected from December 2019 to July 2020. SARS-CoV-2 reference genome (RefSeq: NC_045512.2) was obtained from the GenBank database. Genomes alignment was performed using MAFFT [27].

Nsp1 sequence belonging to SARS-CoV-2 were identified using BioEdit and verified by using BLAST [28]. Nsp1 protein of SARS-CoV-2 with and without deletion have been subsequently modelled using I-TASSER [29].

## Results

**Fig. 1:**
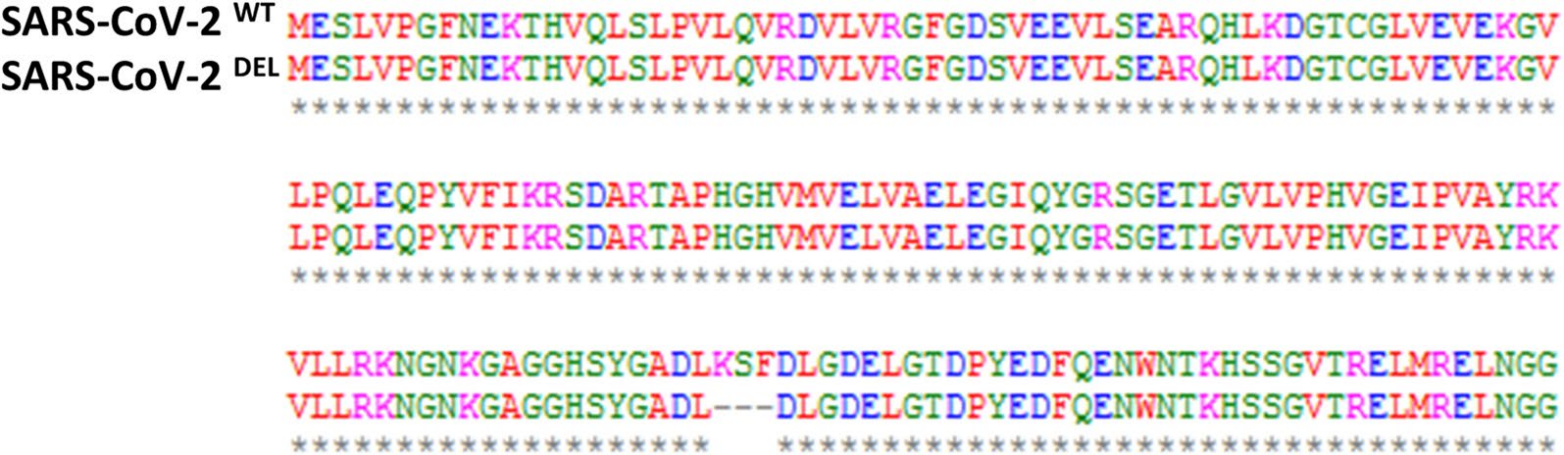
Nsp1 alignment between sequences from SARS-CoV-2 wild type and strains carrying the KSF deletion. The amino acid sequences of SARS-CoV-2 wild type (WT) and SARS-CoV-2 with the 3 amino acids deletion (DEL) were aligned using Clustal Omega. The deletion is shown

We identified genomic sequences, from specific Countries, carrying a deletion of 9 nucleotides in position 686-694, corresponding to AA position 241-243 (KSF) (Fig. 1). The list of Countries with the related number of sequences available analyzed and the number of sequences carrying the deletion is listed in Table 1. The overall presence of genomes carrying the deletion in the cases analyzed was 0.44%, though it was not homogelouly distributed. In fact, we did not found it in certain Countries, such as Italy, Germany and Austria., while in others it was clearly present, for example in Sweden with 10 out of 527 genomes (1.90%), Israel (0.90), Brazil (0.63%) and England (0.45%). Among the States analyzed in the United States, we could detect it in New Jersey (0.91%), New York (0.74), Utah (0.73), and Connecticut (0.65), while we could not detect it in Texas and Nebraska. We note that some of the areas where the deletion could not be detected had a very low number of genomic sequences available for analysis, making the negative results difficult to interpret. Furthermore, the dataset available did not allow us to determine whether this deletion happened as a series of independent events in different temporal moments and geographical areas, as if the virus has an intrisecally fragile site, or it emerged from a single transforming event originating from a unique cluster. More data are needed to differentiate between these hypotheses.

**Table 1:** List of Countries analyzed and number of sequences examined which carry the amino acid deletion

Country	Number of sequences examined	Number of sequences carrying the deletion	Percentage of sequences carrying the deletion
Austria	387	0	0.00
Belgium	754	2	0.27
Brazil	630	4	0.63
Denmark	601	1	0.17
England	8300	37	0.45
France	378	1	0.26
Germany	230	0	0.00
Ireland	16	0	0.00
Israel	222	2	0.90
Italy	146	0	0.00
Netherland	1363	3	2.21
Portugal	501	1	0.22
Spain	1195	2	0.17
Sweden	527	10	1.90
Switzerland	401	0	0.00
Total	15,651	63	0.40
United States			
Utah	275	2	0.73
New York	1345	10	0.74
New Jersey	219	2	0.91
Connecticut	155	1	0.65
Texas	234	0	0.00
Nebraska	49	0	0.00
Total	2277	15	0.66

**Fig. 2:**
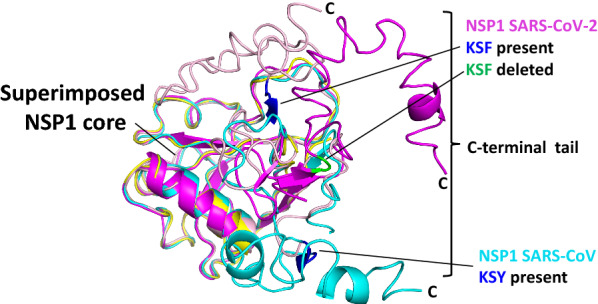
Comparison of NSP1 SARS-CoV and SARS-CoV-2. Comparison of core structure with prediction models of full length nsp1 SARS-CoV (cyan) and SARS-CoV-2 are superimposed in different colors (magenta and light pink). The prediction models for both C-terminal tails of nsp1 SARS-CoV with KSY (blue) and nsp1-SARS-CoV-2 with KSF present (blue) and KSF deleted (green) are predicted to be highly disordered compared with nsp1 Core elements (yellow). R.M.S.D is 0.78Å for core elements. Note that the core structure has been previously resolved for SARS -CoV (PDB code 2GDT), while the C-tail structure has not

We next used I-TASSER to model nsp1 protein of SARS-CoV-2 carrying the deletion. A structure comparison of nsp1 from SARS-CoV-2 models with and without the deletion is represent in Fig. 2. Cartoon depiction of the nsp1 from SARS-CoV-2 with and without the deletion show the superimposed core (AA1-127) and the C-terminal tails (AA128-148) [30]. The structure of the C-terminal tail is unresolved in the NMR structure of SARS-CoV (PDB code 2GDT) and this region is predicted to be highly flexible and disordered, with a few secondary helical elements predicted [31]. Prediction models for both nsp1 SARS-CoV and nsp1 SARS-CoV-2 indicate a possibility of a short helical secondary structure for KSY and KSF amino acids, respectively, and this terminal tail was found to be very important for expression of nsp1 itself [18]. The flexibility, lack of structure and disorder in this region is speculated to allow for availability of the protease recognition seuquence between nsp1 and nsp2 [31]. Indeed, the C-terminal tail was found to be dispensable for MHV (murine hepatitis virus) viral replication but necessary for proteolysis of nsp1 and nsp2 [32]. The newly described deletion of KSF amino acids may influence potential secondary structure in this region of SARS-CoV-2, thereby altering activity of nsp1 interactions and consequent activity on viral protein and host’s gene expression regulation.

## Discussion

Our analysis shows the emergence of a deletion in nsp1, one of the most important determinants of pathogenicity of SARS-CoV-2. This is quite surprising, since corona viruses typically experience a moderate rate of mutations, due to the presence of a protein with proofreader activity (ExonN, also called nsp14), calculated in about 26 mutations per year (https://nextstrain.org/ncov/global?l=clock). Though the number of sequences detected was a small fraction of the total analyzed, our data clearly identify a new SARS-CoV-2 viral strain present in subjects from different areas (Europe, North and South America). However, our analysis also indicates that this deletion is not homogeneously present in all the Countries analyzed. For this reason, it would be important to monitor its presence over time, and to determine its penetrance and probability to spread and compete with the current viral strains. Nonetheless, our results suggest the possibility of the evolution of a new viral quasi-specie, but further data are necessary to confirm this hypothesis and explore the possibility of a developing intra-host adaptative process.

The new viral strain that we describe carries a defining characteristic deletion of 9 nucleotides in the C-terminal region of the nsp1 gene, translating into a protein lacking three amino acids (KSF). Substitution of two of these amino acids (KS) reduced the inhibitory effect of innate immune response to SARS-CoV, and by predicted structure analysis we show that these amino acids compromise proper folding of nsp1. Consequently, we hypothesize that viruses harboring this deletion are likely to be less pathogenic than commonly observed viral strains. To this regard, we note that the two common endemic human coronaviruses, HCoV-OC43 [33] and HCoV-299E [34], have extensive deletions in the C-terminal region of nsp1. Thought crystallization and biological data are needed to confirm our hypothesis, our observations, together with the recent findings of two viral strains carrying in one case an extensive deletion in the orf7a gene [35], a deletion in the nsp2 gene [36] and deletions in nsp1 gene also identified by other groups [37, 38], indicate that SARS-CoV-2 genome may be undergoing a significant evolutionary process, which may result in virus-host adaptation [39]. Since the overwhelming majority of genomic sequences collected so far are from symptomatic subjects, it seems logical to characterize in detail SARS-CoV-2 genomes from the asymptomatic population. If our hypothesis is correct, this is the proper population where we should be able to identify more in detail further viral evolutionary steps, which may indicate reduction of pathogenicity. Understanding the different steps that characterize the pathogenicity of this virus, as well as the spreading and changes of these pathogenic determinants among the population, may help determining proper strategies of containment of SARS-CoV-2 spread and identify better drugs for treatment of COVID-19.

## Conclusions

We identified the emergence in infected subjects of a new viral strain of SARS-CoV-2 with a deletion of 3 amino acids (KSF) in the C-terminal region of nsp1. I-TASSER structure analysis indicates that this deletion may affects the structure of the C-terminal region, important for regulation of nsp1 activity. Substitution of two of these amino acids (KS) was also previously reported to revert the loss of interferon-alpha expression in cells transfected with mutated nsp1 from SARS-CoV. This deletion in nsp1, together with deletions previously described in other parts of SARS-CoV-2 genome by different groups, indicates that the virus is undergoing profound genomic changes. It should be noted that mutations of the virus are not very common, due to its proofreading mechanism, and that collection of the sequencing data is currently biased toward symptomatic subjects. It would be of interest to monitor over time and confirm the spreading of this particular viral strain, and potentially of strains with other deletions in the nsp1 protein, in the population of asymptomatic and pauci-symptomatic subjects and to correlate these changes in nsp1 with a possible decreased viral pathogenicity.

## Data Availability

All data utilized, generated or analyzed during these studies are included in this published article.

## Abbreviations

SARS-CoV: Severe acute respiratory syndrome coronavirus
SARS-CoV-2: Severe acute respiratory syndrome coronavirus 2 (COVID-19)
MERS-CoV: Middle east respiratory syndrome coronavirus
COVID-19: Coronavirus disease-19
